# Photoacoustic imaging of tumour vascular permeability with indocyanine green in a mouse model

**DOI:** 10.1186/s41747-018-0036-7

**Published:** 2018-02-27

**Authors:** Kenichiro Okumura, Kotaro Yoshida, Kazuaki Yoshioka, Sho Aki, Norihide Yoneda, Dai Inoue, Azusa Kitao, Takahiro Ogi, Kazuto Kozaka, Tetsuya Minami, Wataru Koda, Satoshi Kobayashi, Yoh Takuwa, Toshifumi Gabata

**Affiliations:** 1 Department of Radiology, Kanazawa University School of Medical Sciences, 13-1 Takara-machi, Kanazawa, Ishikawa 920-8641 Japan; 2 Department of Physiology, Kanazawa University School of Medical Sciences, Ishikawa, Japan; 3 Department of Quantum Medical Technology, Kanazawa University Graduate School of Medical Sciences, Ishikawa, Japan

**Keywords:** Photoacoustic imaging, Indocyanine green, Vascular permeability, Anti-angionenic treatment

## Abstract

**Background:**

We analysed the haemodynamics of indocyanine green (ICG) in mouse organs and tumours and evaluated responses to anti-angiogenic agents in an allograft tumour mouse model by photoacoustic imaging.

**Methods:**

Thirty-six male mice (aged 10–14 weeks; body weight 20–25 g) were used. Real-time photoacoustic imaging of organs and tumours after intravenous injection of ICG was conducted in mice until 10 min after ICG injection. ICG distribution in tumour tissues was assessed by immunohistochemical staining and observation of ICG-derived fluorescence. Vascular permeability changes induced by the vascular endothelial growth factor (VEGF)-blocking agent VEGF-trap on tumour photoacoustic signals were studied.

**Results:**

The photoacoustic signals in salivary glands and tumours after intravenous injection of iCG (0.604 ± 0.011 and 0.994 ± 0.175 [mean ± standard deviation], respectively) were significantly increased compared with those in the liver, kidney, and great vessel (0.234 ± 0.043, 0.204 ± 0.058 and 0.127 ± 0.040, respectively; *p* < 0.010). In tumours, the photoacoustic signal increased within 30 s after ICG injection in a dose-dependent manner (r^2^ = 0.899) and then decreased gradually. ICG was found to extravasate in tumour tissues. In VEGF-trap-treated mice, the photoacoustic signal in the tumour decreased at the early phase before inhibition of tumour growth was detected (0.297 ± 0.052 vs 1.011 ± 0.170 in the control; *p* < 0.001).

**Conclusions:**

Photoacoustic imaging with ICG administration demonstrated extravasation of ICG in mouse organs and tumours, indicating the potential for early detection of changes in vascular permeability during cancer therapy.

## Key points

- The photoacoustic signal of indocyanine green was increased in tumours
- Indocyanine green was found to extravasate in tumour tissues
- The photoacoustic signal was derived from extravasated indocyanine green
- In VEGF-trap-treated mice, photoacoustic signals decreased before tumour shrinkage

## Background

Photoacoustic imaging is an emerging non-invasive optical technology that proved to have potential for cellular and molecular specific visualisation at clinically relevant depths of various organs [1]. As apparatuses for photoacoustic imaging are commercially available only for animal experiments or preclinical studies, a few clinical trials have been undertaken based on the promising results obtained in the former [2]. Some endogenous chromophores present in the body, such as melanin and haemoglobin serve as target optical absorbers for photoacoustic imaging [3]. Moreover, exogenous molecular imaging agents which can be used as contrast agents for various imaging technologies (e.g. metallic nanostructures, carbon nanotubes, and fluorescence dyes) have been found to be applicable to photoacoustic imaging [4].

Indocyanine green (ICG) is a fluorescent cyanine dye used as a medical diagnostic agent for monitoring cardiac and liver function [5, 6]. ICG has also been used as a near-infrared (NIR) chromophore to visualise the target structures of organs during intraoperative microscopy coupled with a charge-coupled device camera, enabling real-time NIR imaging in vivo [7–9]. However, NIR imaging combined with ICG is clinically limited during open or endoscopic surgery due to the limited penetration depth of NIR (3–5 mm) [10]. Although bioluminescence imaging is a non-invasive modality with considerable potential for visualisation in deeper tissues and is widely used in the field of preclinical animal research, those studies have been limited by difficulties in non-invasively monitoring early disease progression in real time with high spatial resolution [11].

In this study, the potential of ICG-enhanced photoacoustic imaging was evaluated using organs and tumour allografts from mice. Moreover, the applicability of photoacoustic imaging coupled with ICG for evaluating changes in tumour vascular permeability associated with anti-angiogenic therapy was examined. The purpose of this study was to analyse the haemodynamics of ICG in mouse organs and tumours and evaluate the tumour response to anti-angiogenic agents by photoacoustic imaging.

## Methods

### Ethical considerations

All procedures were conducted in accordance with the Fundamental Guidelines for Proper Conduct of Animal Experiment and Related Activities in Academic Research Institutions under the jurisdiction of the of the Ministry of Education, Culture, Sports, Science and Technology of Japan and approved by the Committee on Animal Experimentation of Kanazawa University.

### Photoacoustic imaging system

**Fig. 1:**
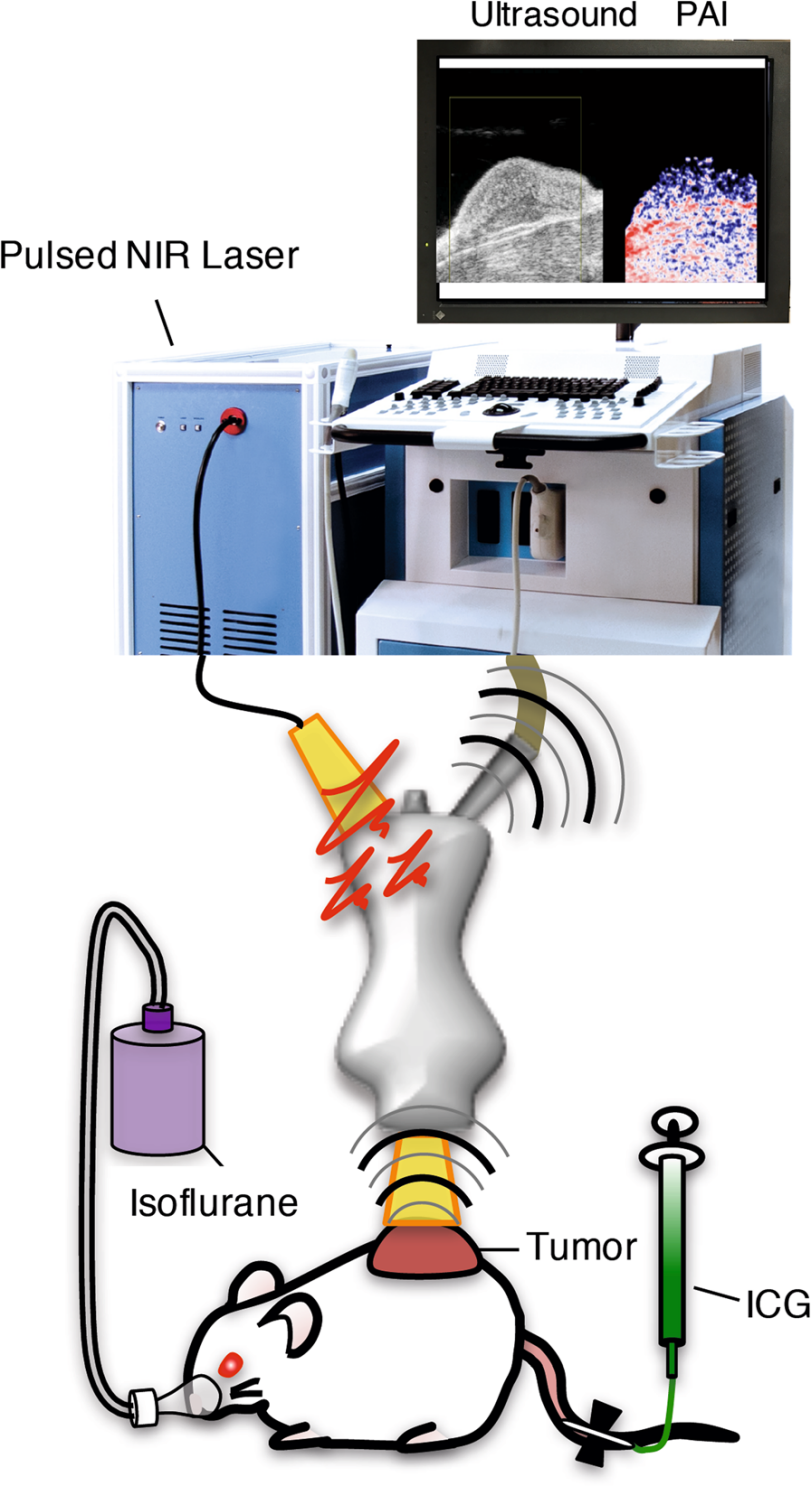
Schematic diagram of the photoacoustic imaging system. Mice were under inhalational anaesthesia (isoflurane, 2% in room air); ICG was injected via the tail vein. The pressure transients produced from chromophores in tissues were scanned by an ultrasound transducer when the tissue was exposed to a pulsed NIR laser

Photoacoustic imaging was carried out with a Vevo LAZR small-animal ultrasound and photoacoustic imaging system (Visualsonics, Toronto, ON, Canada). This machine was composed of an ultrasound system equipped with a laser pulse system and animal measurement box (Fig. 1). To acquire photoacoustic images, a LZ-550 linear-array transducer (256 elements, 40-MHz centre frequency, 27-MHz bandwidth) was used. The transducer was connected to both the ultrasound and laser system, with a pulsed laser beam illuminating the tissue from the probe tip. The tuneable laser in this system supplied 10–20 mJ/pulse across its 680–970-nm wavelength range, with a pulse repetition frequency of 10 Hz. All acquired images were stored on a dedicated purpose computer, a region of interest was placed on the target organ or tumour, and the photoacoustic signals of the region of interest were recorded.

### In vitro experiments

To investigate the reliability and quantitative capacity of photoacoustic imaging, in vitro experiments were performed. Three different concentrations of ICG (1, 2.5 and 5 mg/ml Diagnogreen; Daiichi Sankyo, Tokyo, Japan) and saline as a control were imaged on cotton buds and measured using the photoacoustic imaging system.

### In vivo animal experiments

For in vivo experiments, C57BL6/J and B6N-Tyr^c-Brd^/BrdCrCrl albino (SLC, Japan) male mice (aged 10–14 weeks; body weight, 20–25 g) were used. Hair at the examination site was removed with hair removal cream one day before the photoacoustic imaging procedure.

A Lewis lung carcinoma (LLC) cell allograft model was used. Briefly, LLC cells were cultured in minimum essential medium supplemented with 10% foetal bovine serum. At 70–80% confluence, the tumour cells were trypsinised and 1.0 × 10^7^ cells suspended in 100 μL were injected subcutaneously into the backs of the mice under general anaesthesia with pentobarbital (30 mg/kg, intraperitoneally). Subcutaneous tumours were imaged with a photoacoustic imaging system ten days after tumour implantation, when the tumours had reached about 1 cm in maximum diameter.

**Fig. 2:**
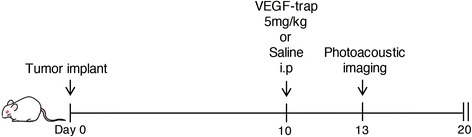
Experimental design in a tumour mouse model with anti-VEGF therapy. Each mouse was given one intraperitoneal injection of VEGF-trap ten days after implantation of LLC into the subcutaneous tissue. Photoacoustic imaging was performed after three days and we evaluated the tumour volume 20 days later

For anti-vascular endothelial growth factor (VEGF) antibody treatment, mice were injected intraperitoneally with VEGF-trap (Regeneron, Tarrytown, NY, USA and Bayer Health Care, Berlin, Germany) at a concentration of 5 mg/kg ten days after tumour implantation. Photoacoustic imaging was performed three days after VEGF-trap injection. Tumour volumes were monitored every two days after tumour implantation for 20 days (Fig. 2). Tumour size was measured by callipers (length and width) every two days. The tumour volume (V = 1/2 length × width^2^) was also calculated.

### Photoacoustic imaging acquisition in mice

For photoacoustic imaging, mice were anesthetised with isoflurane (2.0%) and fixed on a platform. Each target organ was observed by B-mode ultrasound and a favourable position at maximum area coverage was chosen. Before injection of ICG, pre-contrast photoacoustic imaging was performed for 1 min. Next, ICG was injected via the tail vein using a 27-G butterfly needle attached to a disposable microsyringe at an initial rate of 0.02 ml/s using an automatic injector (Fusion Touch, ISIS, Osaka, Japan). Signals of photoacoustic imaging were obtained under the following conditions: 780 nm excitation light; 42-dB photoacoustic gain; 22-dB two-dimensional gain and 5 frames/s. To reduce the effects of respiratory motion, we used a respiratory gate. Photoacoustic signals in the liver, kidney, salivary gland, inferior vena cava and LLC implanted in the skin of mice were collected during the 10-min period after intravenous ICG injection (5 mg/ml) and peak photoacoustic signals were calculated. For semi-quantitative analysis of tumours after ICG injection, three different concentrations of ICG (1, 2.5 and 5 mg/ml) were administered and photoacoustic signals were acquired. For experiments involving VEGF-trap treatment, 5 mg/ml ICG was administered and peak photoacoustic signals were calculated. Photoacoustic signals were acquired during the 10-min period until the signal reached a plateau.

### Histology

To visualise the extravasation of ICG in tumour tissues, ICG was injected via the tail vein; 30 s later, vessels were washed with saline under cardiac puncture and drained from the superior vena cava. Tumour tissues were embedded in Optical Cutting Temperature compound (Tissue-Tek, Sakura Finetek). Cryosections (10 μm) were stained with anti-CD31 antibodies (1:400; cat. no. 550274; BD) overnight at 4 °C and incubated with Alexa Fluor 488-conjugated secondary antibodies (A11034; Molecular Probes, Eugene, OR, USA) for 1 h at room temperature. Cell nuclei were counterstained with 4′,6-diamidino-2-phenylindole (cat. no. D1306; Molecular Probes). Samples were imaged using an inverted microscope (model IX70; Olympus, Tokyo, Japan) equipped with an ORCA-Flash4.0 digital CMOS camera (Hamamatsu Photonics), configured with a ICG-filter set (ICG-B-U02; Semrock) or with confocal laser microscopy, as previously described [12].

### Statistical analysis

Statistical analysis and graphical display of data were performed using GraphPad software (version 7.00 for Mac; GraphPad Software, San Diego, CA, USA). All values are reported as mean ± standard deviation (SD). One-way analysis of variance with post-hoc Tukey test was used to compare photoacoustic signals among groups. Correlation analysis between photoacoustic signals and ICG concentrations in vitro experiments and between photoacoustic signals in tumours and ICG concentrations in the animal experiment was performed using Pearson’s correlation analysis. Two-tailed unpaired t-tests were used to compare photoacoustic signals and tumour volumes between the control and anti-VEGF therapy groups. Results with *p* values < 0.050 were considered as statistically significant.

## Results

### In vitro experiment

**Fig. 3:**
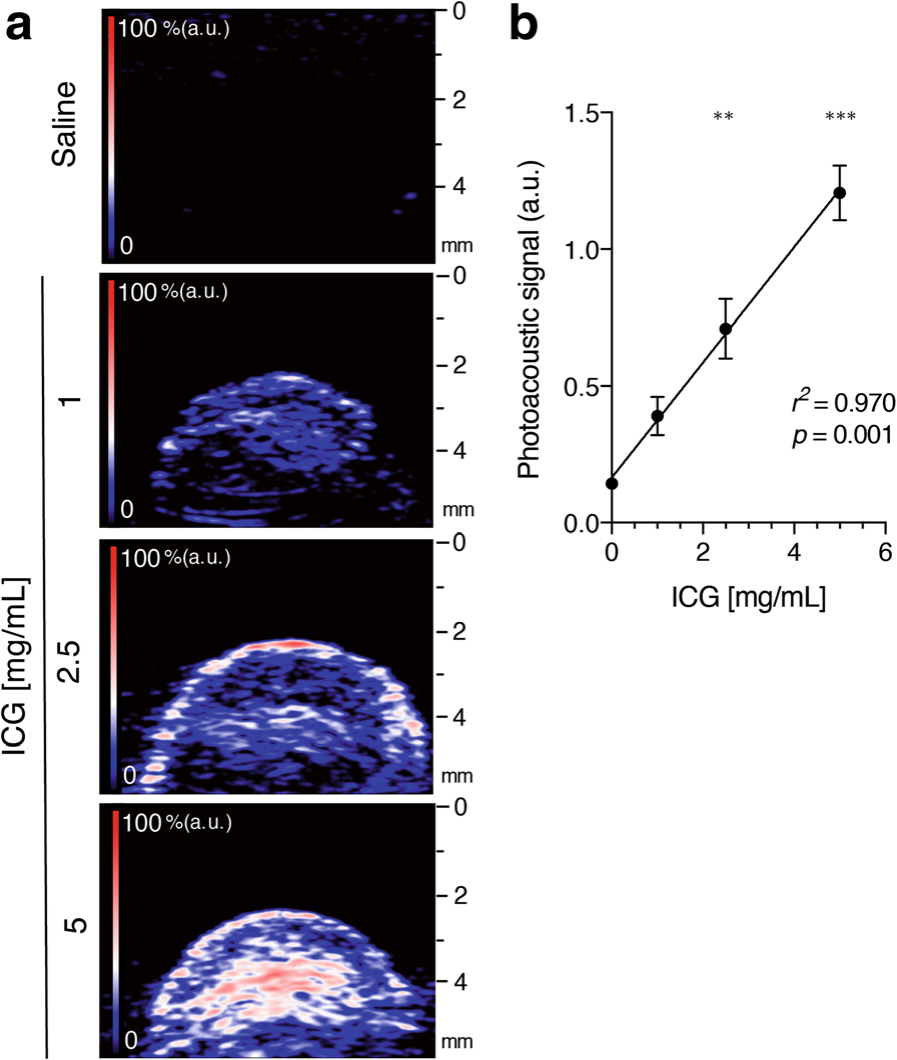
In vitro photoacoustic imaging of ICG. **a** Representative images showing a map of optical absorption from ICG in cotton swabs soaked with the indicated concentrations of ICG (0, 1, 2.5 and 5 mg/ml). Brighter signals in the photoacoustic imaging map corresponded to higher optical absorption of ICG chromophores. The *colour map* has *red graduation* that fades into *blue* (n = 4). **b** *Graph* showing the linear relationship between the mean peak photoacoustic signal (mean ± SD) and ICG dose. The regression line shows a significant correlation with an r^2^ value of 0.970 (n = 4)

Cotton swabs presoaked with three different concentrations of ICG provided dose-dependent increases in photoacoustic signals (Fig. 3a), demonstrating a linear relationship between the ICG dose and the photoacoustic signal intensity (r^2^ = 0.970; Fig. 3b, Table 1).

**Table 1:** ICG-derived photoacoustic on different concentration

ICG concentration (mg/ml)	Photoacoustic signal (a.u.)
0.0	0.143 ± 0.014
1.0	0.391 ± 0.070
2.5	0.710 ± 0.109^a^
5.0	1.206 ± 0.101^b^

Data are means ± SD. a.u. = arbitrary units

^a^Significant difference compared to ICG concentration 1.0 mg/ml (*p* < 0.010)

^b^Significant difference compared to ICG concentration 1.0 and 2.5 mg/ml (*p* < 0.001)

### ICG-photoacoustic imaging in mice

**Fig. 4:**
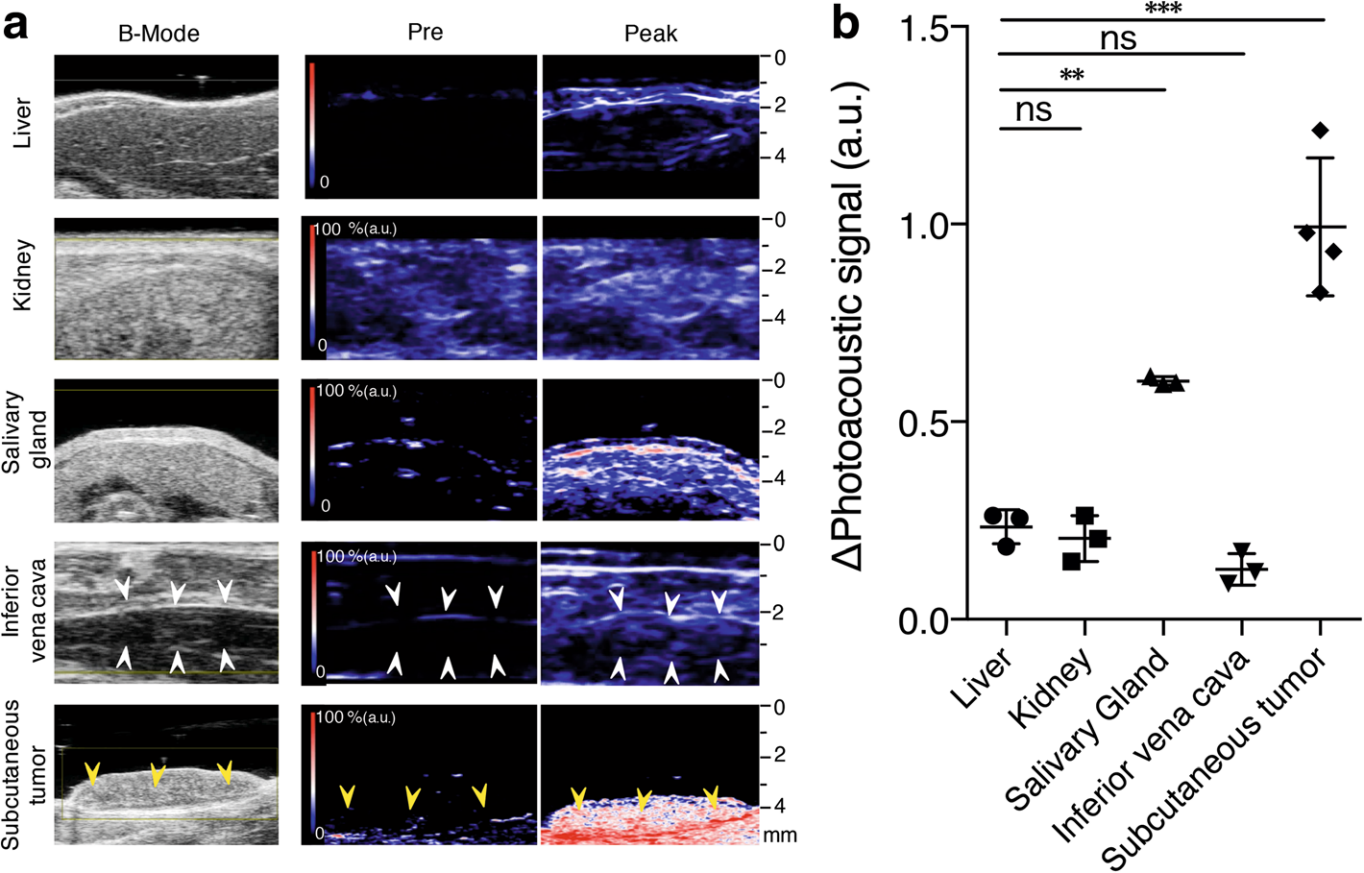
Photoacoustic imaging with various organs and allograft tumours in mouse skin. **a** Representative co-registered ultrasound B-mode and photoacoustic imaging of various organs (liver, kidney, salivary gland and inferior vena cava) and subcutaneous Lewis lung carcinoma (LLC) allograft tumour in mice 30 s after intravenous injection of ICG (5 mg/ml). *White arrow-head*: blood vessel, *yellow arrow head*: subcutaneous LLC tumour burden with strong photoacoustic signal. **b** *Graph* showing the maximum photoacoustic signal (mean and SD) of organs and subcutaneous LLC tumours in mice after intravenous injection of ICG (5 mg/ml; n = 3 or 4). ns not significant. **Significant difference compared with the liver, kidney and inferior vena cava. ***Significant difference compared with the liver, kidney, inferior vena cava and salivary gland

In all examined organs photoacoustic signals rapidly increased after ICG injection and peaked within approximately 30 s, followed by gradual decay (Fig. 4a). The quantification of the peak photoacoustic signals demonstrated that the photoacoustic signal in tumours was significantly higher than those in the salivary gland, liver and kidney in ICG-injected mice (Fig. 4b, Table 2). The photoacoustic signal in the blood-filled inferior vena cava was weaker than that in other organs, whereas tumours exhibited the highest photoacoustic signals (Fig. 4b).

**Table 2:** ICG-enhanced photoacoustic signals in organs and tumours

	Signal increase (a.u.)
Liver	0.234 ± 0.043
Kidney	0.204 ± 0.058
Salivary gland	0.604 ± 0.011^a^
Inferior vena cava	0.127 ± 0.040
Tumour	0.994 ± 0.175^b^

Data are means ± SD. a.u. = arbitrary units

^a^Significant difference compared with liver, kidney and inferior vena cava (*p* < 0.010 [to liver and kidney], *p* < 0.001 [to IVC])

^b^Significant difference compared to liver, kidney, salivary gland and inferior vena cava (*p* < 0.010 [to salivary gland], *p* < 0.001 [to liver, kidney and inferior vena cava])

### Enhanced ICG-injection-induced photoacoustic signals in tumours

**Fig. 5:**
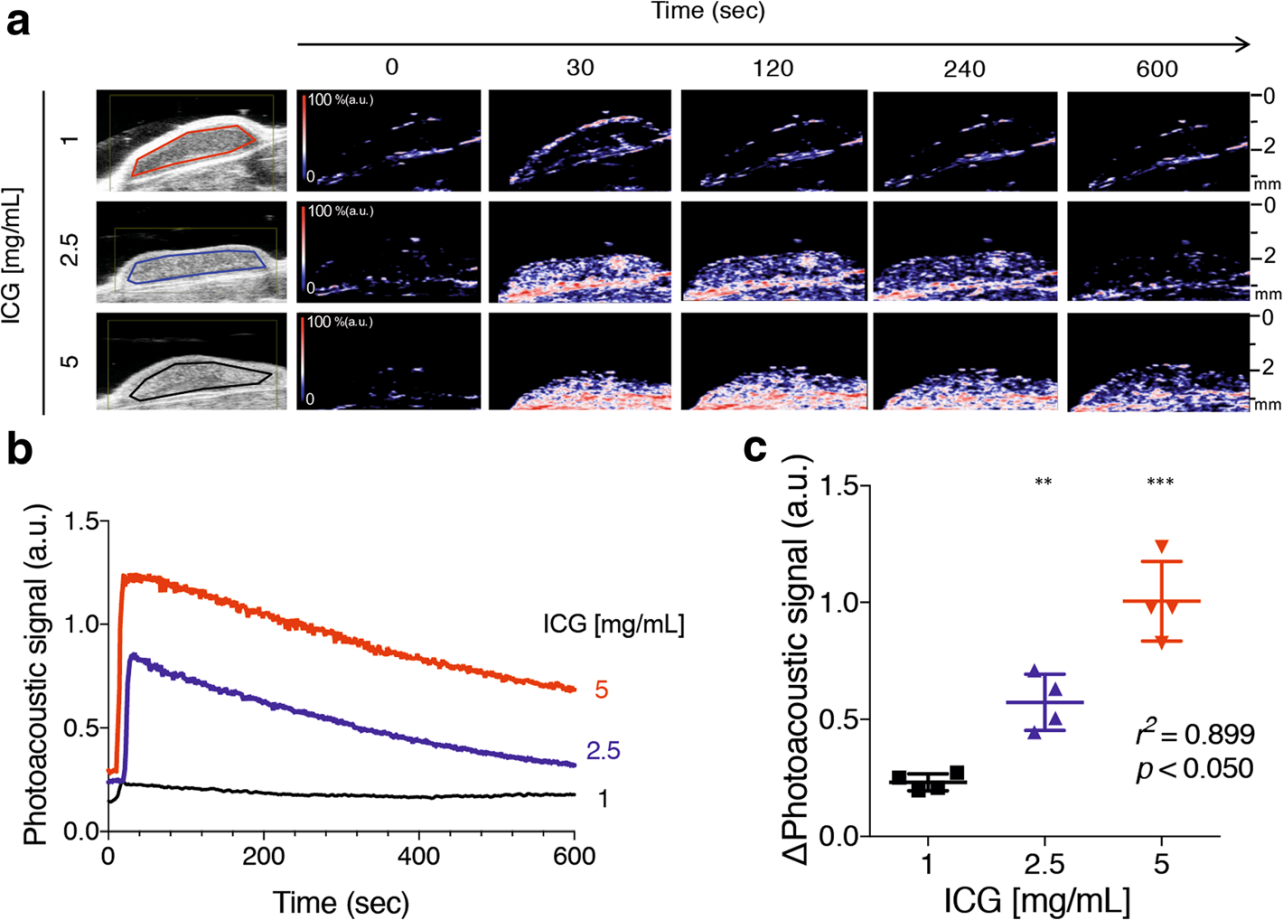
Photoacoustic imaging of ICG dynamics in subcutaneous mouse tumours. **a** Representative images of co-registered ultrasound B-mode and photoacoustic imaging showing the LLC tumour at different times after intravenous injection of the indicated ICG dose (1, 2.5 and 5 mg/ml). **b** *Trace graph* showing serial measurements of photoacoustic imaging in subcutaneous LLC tumours after intravenous injection of different ICG doses (1, 2.5 and 5 mg/ml; n = 4). **c** *Graph* showing the maximum photoacoustic signals (mean and SD) of subcutaneous LLC tumours in mice (n = 4). **p < 0.010, ***p < 0.001

B-mode photoacoustic imaging showed increases in photoacoustic signals diffusely within tumours after ICG injection (Fig. 5a). The photoacoustic signals in tumours appeared to be inhomogeneous and granular. Quantitatively, the photoacoustic signal immediately rose just after ICG injection, reaching a peak by 30 s, and then showed a gradual decline (Fig. 5b and c). The increase in photoacoustic signal after ICG injection was clearly dependent on the amount of ICG injected (r^2^ = 0.899; Fig. 5b and c, Table 3).

**Table 3:** ICG-enhanced photoacoustic signals of tumours at different concentrations

ICG concentration (mg/ml)	Signal increase (a.u.)
1.0	0.231 ± 0.036
2.5	0.574 ± 0.120^a^
5.0	1.005 ± 0.171^b^

Data are means ± SD. a.u.= arbitrary units

^a^Significant difference compared with ICG concentration 1.0 mg/ml (*p* < 0.010)

^b^Significant difference compared with ICG concentration 1.0 and 2.5 mg/ml (*p* < 0.001 [to 1.0], *p* < 0.010 [to 2.5])

**Fig. 6:**
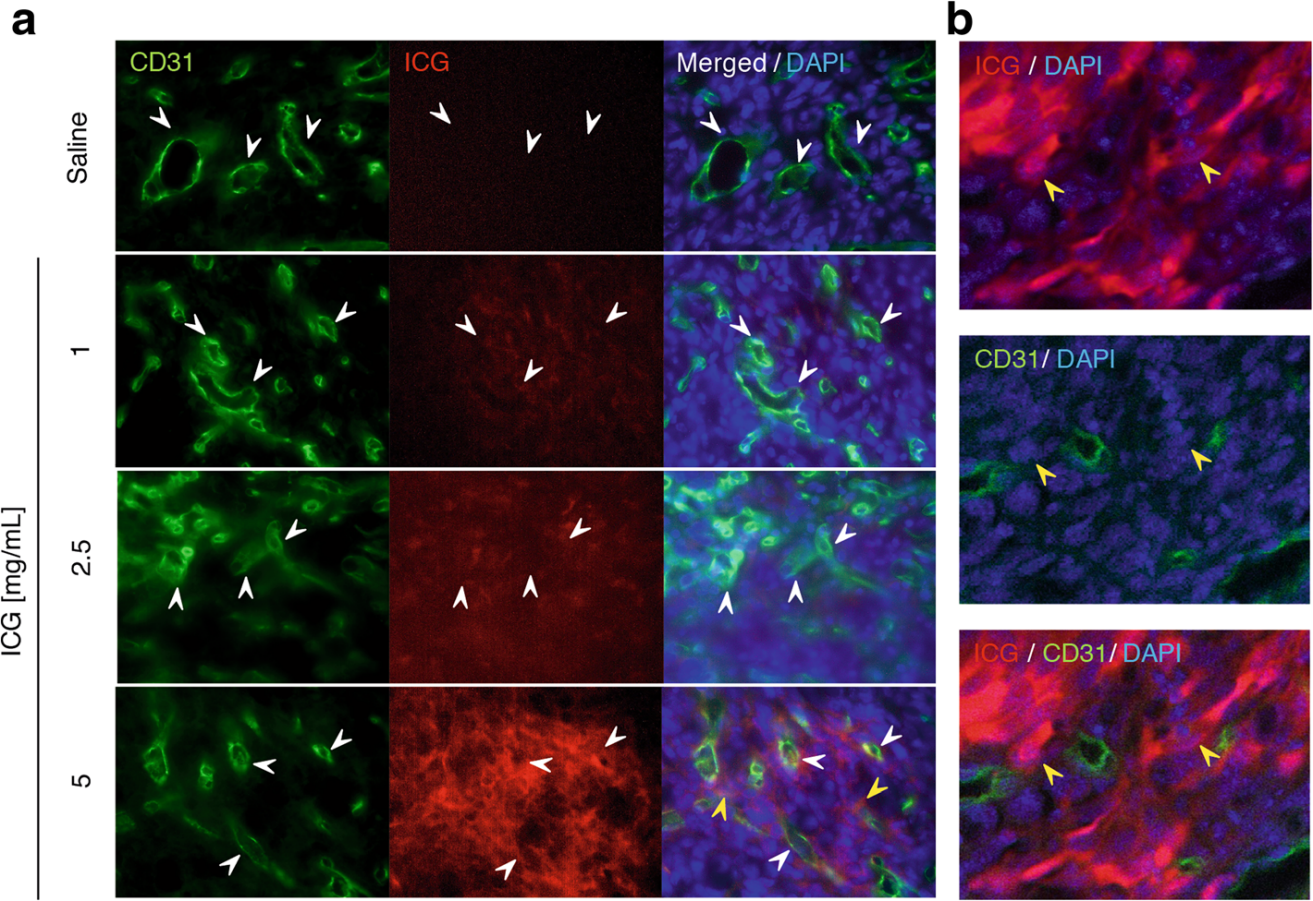
Extravasation of ICG into the interstitial space surrounding tumour vasculature. **a** Immunofluorescence staining (original magnification, 40×) of LLC tumour sections demonstrating ICG accumulation at different doses (1, 2.5 and 5 mg/ml; *pseudo-red colour*) surrounding CD31^+^ tumour capillaries (*green*, *white arrowheads*) of LLC tumours 30 s after intravenous administration. Nuclei were counterstained with 4′,6-diamidino-2-phenylindole (*blue*). Scale bar: 20 μm. **b** Confocal fluorescence imaging of LLC tumour sections (original magnification, 60×). Note that ICG (5 mg/ml) was distributed extensively in the tumour cells, but not in CD31^+^ tumour capillaries (*yellow arrowheads*)

On histopathological examination, extravascular leakage of ICG was observed in the interstitial space outside the vascular lumen lined by CD31-positive vascular endothelium in tumours (Fig. 6a). Extravascular ICG was greater in mice injected with a high dose of ICG compared with that in mice injected with low and medium doses of ICG (Fig. 6a). Moreover, the cells in the extravascular space, most likely tumour cells, appeared to take up and accumulate ICG within cells, as observed with a confocal laser microscope (Fig. 6b).

### Normalisation of ICG-photoacoustic signals in tumours by a VEGF-blocking agent

**Fig. 7:**
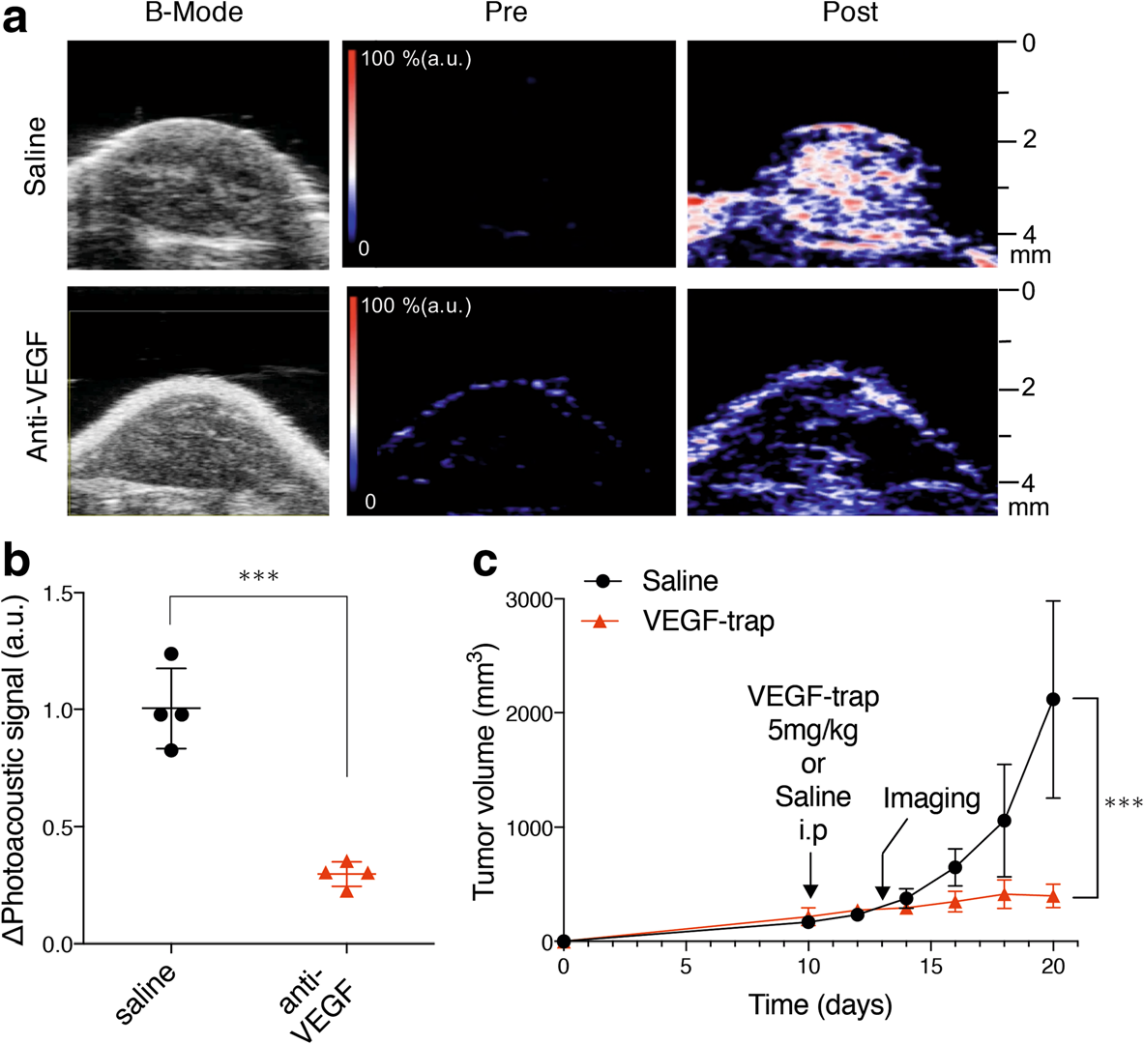
Normalisation of ICG-photoacoustic signals in tumours with a VEGF-blocking agent. **a** Representative photoacoustic imaging with B-mode images show prevention of ICG vascular leakage in subcutaneous LLC tumours with anti-VEGF therapy. The *colour map* has *red gradation* that fades into *blue* (n = 4, ICG: 5 mg/ml). **b** *Graph* showing maximum photoacoustic signal values of subcutaneous LLC tumours with or without anti-VEGF therapy (*p* < 0.001; n = 4, ICG: 5 mg/ml). **c** *Growth curves* of subcutaneous LLC tumours in mice treated with control saline (*black circle*) and VEGF-trap (*red triangle*, 5 mg/kg). Data shown are means ± SD. Treatment and imaging schedules are indicated with *arrows* (n = 4, ICG 5 mg/ml). ***p < 0.001

A single administration of VEGF-trap dramatically decreased ICG-photoacoustic signals on day 13 compared with saline administration (Fig. 7a and 7b, Table 4), although VEGF-trap did not alter tumour volumes at that time point (Fig. 7c). Administration of VEGF-trap caused a decrease in the tumour volume from day 16 (six days after VEGF-trap administration) and induced an approximate 80% reduction in tumour volume on day 20 compared with saline administration (Fig. 7c, Table 4), confirming the anti-tumour effects of VEGF-trap.

**Table 4:** ICG-enhanced photoacoustic signals of tumours and changes in tumour volumes with anti-VEGF therapy

	Signal increase (a.u.)	Tumour volume on day 20 (mm^3^)
Saline	1.011 ± 0.170	2117.680 ± 864.375
Anti-VEGF	0.297 ± 0.052^a^	397.753 ± 102.773^a^

Data are means ± SD. a.u.= arbitrary units

^a^Significant difference compared to control (*p* < 0.001)

## Discussion

This study showed that photoacoustic imaging with ICG administration could be used to evaluate ICG extravasation in tumours and facilitate the early detection of changes in vascular permeability during cancer therapy. The use of the photoacoustic imaging system enabled safe and real-time image acquisition to convey underlying pathologic information for the target tissue without radiation exposure.

Some experimental studies and preclinical studies using photoacoustic imaging have been reported, including non-invasive imaging of the breast, sentinel lymph nodes and skin [13–16]. However, photoacoustic imaging is still limited by its inability to non-invasively characterise tumour development in the preclinical and clinical settings. To date, ICG is thought to be a suitable exogenous molecular imaging agent for photoacoustic imaging. ICG accumulation in sentinel lymph nodes, breast cancer and liver tumours, such as hepatocellular carcinoma, cholangiocarcinoma and metastatic liver tumours, has previously been visualised by photoacoustic imaging [17]. However, most studies have relied on static measurements, thus lacking dynamic information. Non-invasive dynamic photoacoustic imaging of acute changes in ICG signals after intravenous injection has not been extensively investigated.

In our study, we clarified that ICG can be used as a contrast agent for non-invasive dynamic photoacoustic imaging in a mouse model. Importantly, transient ICG signals in photoacoustic imaging are thought to originate from the extravascular space. Of the various organs tested in this study, photoacoustic signals from tumours and salivary glands showed the strongest peak just after injection. In contrast, the inferior vena cava, including the large intravascular space with flowing blood, did not show any clear increase in ICG signals. This suggested that the ICG signal observed during the early phase after administration was mainly derived from the extravascular space, not the intravascular space with our photoacoustic settings. This observation was supported by data showing that salivary glands containing hyperpermeable fenestrated capillaries revealed a higher photoacoustic signal intensity than other normal organs [18].

ICG diffuses into the systemic vasculature and the ICG bound to albumin is then exclusively metabolised and cleared by the liver at a half-life of about 5 min [19]. However, the dynamics of ICG in the acute phase of photoacoustic have not been elucidated. In this study, in the vascular space, the target agent may shuffle and pass quickly out through the observation window, making it difficult to obtain the signal. The photoacoustic signal of ICG was higher in tumours than in other organs, such as the liver and kidney. ICG rapidly binds to albumin (molecular weight ~ 60 kDa) in plasma [20]. Such macromolecules with a molecular weight > 20 kDa are not able to permeate the endothelium of normal organs. In contrast, tumour microvessels with discontinuous endothelium enable larger molecules (> 50 kDa) to extravasate into the interstitial space [21, 22]. The photoacoustic signal of ICG reflects extravasation of ICG-bound albumin accumulation into the extravascular space, followed by vascular hyperpermeability in tumours. To directly confirm ICG extravasation into the interstitial space, we performed immunohistochemical analysis of tumour sections. Immunofluorescence staining of tumour microvessels with anti-CD31 antibodies revealed that ICG accumulated not only in the interstitial space but also in tumour cells. Consistent with our observations, Onda et al. [23] recently demonstrated that intravenously administered ICG was passively internalised by the endocytic activity of tumour cells and entrapped in the membrane trafficking system, resulting in its slow turnover and prolonged retention by tumours.

The objective tumour response has been used as an important endpoint for assessment of the therapeutic effects of cancer therapy. With molecular targeted drugs, including VEGF inhibitors, tumour shrinkage is delayed and tumour cells may disappear without any reduction in the tumour volume. When using molecular targeted drugs, downregulation of VEGF, normalisation of the tumour vasculature, pruning of excess vessels and reduction of interstitial fluid pressure and vessel permeability occur in the tumour consecutively [24–29]. In this study, photoacoustic imaging coupled with ICG revealed that VEGF-trap effects could be predicted in terms of tumour permeability before tumour shrinkage by VEGF-trap therapy.

In conclusion, we found that photoacoustic imaging with ICG administration demonstrated extravasation of ICG in mouse organs and tumours, indicating the potential for early detection of changes in vascular permeability during cancer therapy.

## Abbreviations

ICG: Indocyanine green
LLC: Lewis lung carcinoma
NIR: Near-infrared
VEGF: Vascular endothelial growth factor
